# A quadruple blinded placebo controlled randomised trial to evaluate the effectiveness of an Iodine complex for patients with mild to moderate COVID-19 in Pakistan (I-COVID-PK): A structured summary of a study protocol for a randomised controlled trial

**DOI:** 10.1186/s13063-021-05081-3

**Published:** 2021-02-10

**Authors:** Sohaib Ashraf, Shoaib Ashraf, Moneeb Ashraf, Muhammad Ahmad Imran, Larab Kalsoom, Uzma Nasim Siddiqui, Iqra Farooq, Muhammad Ghufran, Romaisa Arshad Khokhar, Muhammad Kiwan Akram, Iqra Shahid, Muhammad Sohaib Ur Rehman, Rutaba Akmal, Arbaz Tahir, Ayesha Zahid, Sidra Ashraf, Sundas Rafique, Nazish Matti, Muhammad Faisal Nadeem, Ayesha Humayun, Amber Malik, Qazi Abdul Saboor, Ali Ahmad, Muhammad Ashraf, Mateen Izhar

**Affiliations:** 1 Department of Cardiology, Shaikh Zayed Post-Graduate Medical Institute, Lahore, Pakistan; 2 Department of Pathobiology, Riphah International, Lahore, Pakistan; 3 Department of Pharmacology, Mayo Hospital, King Edward Medical University, Lahore, Pakistan; 4 Department of Microbiology, Shaikh Zayed Post-Graduate Medical Institute, Lahore, Pakistan; 5 Department of Medicine, Services Institute of Medical Sciences, Lahore, Pakistan; 6 Department of Medicine, Shaikh Zayed Post-Graduate Medical Institute, Lahore, Pakistan; 7 Department of Medicine, Port Macquarie Base Hospital, New south Wales, Australia; 8 Department of Paediatric Surgery, Children Hospital, Lahore, Pakistan; 9 ESACHS (Empresa de Servicios Externos de la Asociación Chilena de Seguridad), Santiago, Chile; 10 Department of Oral Pathology, Shifa College of dentistry, Shifa Tameer-e-Millat University, Islamabad, Pakistan; 11 Department of animal nutrition, University of veterinary and animal sciences, Lahore, Pakistan; 12 Department of gynaecology and obstetrics, Benazir Bhutto Hospital, Rawalpindi, Pakistan; 13 Department of Public Health and Community Medicine, Shaikh Zayed Medical complex, Lahore, Pakistan; 14 Department of Community Medicine, Sahara Medical College, Narowal, Pakistan; 15 Department of Community Medicine, Faisalabad Medical University, Faisalabad, Pakistan; 16 Department of biochemistry, University of veterinary and animal sciences, Lahore, Pakistan; 17 Department of West Medicine, Mayo Hospital, King Edward Medical University, Lahore, Pakistan; 18 Department of Pharmacy, Quaid-i-Azam University, Islamabad, Pakistan; 19 Institute of Pharmaceutical Sciences, University of Veterinary and Animal Sciences, Lahore, Pakistan; 20 Department of Cardiology, Evercare Hospital, Lahore, Pakistan; 21 Department of Microbiology, Infectiology and Immunology, Centre Hospitalier Universitaire (CHU) Sainte Justin/University of Montreal, Montreal, Canada; 22 Department of Pharmacology and Toxicology, University of veterinary and animal sciences, Lahore, Pakistan

**Keywords:** Iodine, Pakistan, COVID-19, Randomised controlled trial, Protocol

## Abstract

**Objectives:**

The objective of the study is to measure the efficacy of ionic-iodine polymer complex [1] for clinical and radiological improvement in coronavirus disease 2019 (COVID-19) patients.

**Trial design:**

The trial will be closed label, randomized and placebo-controlled with a 1:1:1:1 allocation ratio and superiority framework.

**Participants:**

All PCR confirmed COVID-19 adult patients including non-pregnant females, with mild to moderate disease, will be enrolled from Shaikh Zayed Post-Graduate Medical Complex, Ali Clinic and Doctors Lounge in Lahore (Pakistan). Patients with any pre-existing chronic illness will be excluded from the study.

**Intervention and comparator:**

In this multi-armed study ionic-iodine polymer complex with 200 mg of elemental iodine will be given using three formulations to evaluate efficacy. Patients will be receiving either encapsulated iodine complex of 200 mg (arm A), iodine complex syrup form 40 ml (arm B), iodine complex throat spray of 2 puffs (arm C) or empty capsule (arm D) as placebo; all three times a day. All the 4 arms will be receiving standard care as per version 3.0 of the clinical management guidelines for COVID-19 established by the Ministry of National Health Services of Pakistan.

**Main outcomes:**

Primary outcomes will be viral clearance with radiological and clinical improvement. SARS-CoV-2 RT-PCR and HRCT chest scans will be done on the admission day and then after every fourth day for 12 days or till the symptoms are resolved. RT-PCR will only be shown as positive or negative while HRCT chest scoring will be done depending on the area and severity of lung involvement [2]. Time taken for the alleviation of symptoms will be calculated by the number of days the patient remained symptomatic. 30-day mortality will be considered as a secondary outcome.

**Randomisation:**

Stratification for initial COVID-19 status (or days from initial symptoms as a proxy), age groups, gender, baseline severity of symptoms and co-morbidities will be used to ensure that the study arms remain balanced in size for the 1:1:1:1 allocation ratio. Randomization will be done using the lottery method. As patients are being admitted at different times, they will be recruited after obtaining their voluntary written informed consent following all standard protocols of the infection, control and disinfection.

**Blinding (masking):**

This is a quadruple (participants, care providers, investigators and outcomes assessors) blinded study where only the study’s Primary Investigator will have information about the arms and their interventions.

**Numbers to be randomised (sample size):**

200 patients will be randomized into four groups with three experimental and one placebo arm.

**Trial Status:**

Protocol Version Number is 2.3 and it is approved from IRB Shaikh Zayed Hospital with ID SZMC/IRB/Internal0056/2020 on July 14^th^, 2020. The recruitment is in progress. It was started on July 30, 2020, and the estimated end date for the trial is August 15, 2021.

**Trial registration:**

Clinical Trial has been retrospectively registered on www.clinicaltrials.gov with registration ID NCT04473261 dated July 16, 2020.

**Full protocol:**

The full protocol is attached as an additional file, accessible from the Trials website (Additional file 1). With the intention of expediting dissemination of this trial, the conventional formatting has been eliminated; this Letter serves as a summary of the key elements of the full protocol. The study protocol has been reported in accordance with the Standard Protocol Items: Recommendations for Clinical Interventional Trials (SPIRIT) guidelines.

**Supplementary Information:**

The online version contains supplementary material available at 10.1186/s13063-021-05081-3.

## Supplementary Information

**Additional file 1.**

## Data Availability

Dr. Sohaib Ashraf will have access to the final trial dataset and this could be available from the author on reasonable request but the dataset is subject to data protection regulations. (email address: sohaib@skzmdc.edu.pk Mobile Number: +1 (857) 316 7995)
