# Supplementary material for: A quadruple blinded placebo controlled randomised trial to evaluate the effectiveness of an Iodine complex for patients with mild to moderate COVID-19 in Pakistan (I-COVID-PK): A structured summary of a study protocol for a randomised controlled trial
Source: Trials. 2021 Feb 10;22:127. doi: 10.1186/s13063-021-05081-3 (PMC7873514; doi:10.1186/s13063-021-05081-3)
Supplement: Supplementary file 1 — Additional file 1. [file 13063_2021_5081_MOESM1_ESM.docx]

**Title**

A quadruple blinded placebo controlled randomised trial to evaluate the effectiveness of the Iodine complex for patients with mild to moderate COVID-19 in Pakistan (I-COVID-PK): A structured summary of a study protocol for a randomised controlled trial.

**Study Principal Investigators:**

1. **Muhammad Ashraf, DVM, PhD (University of Minnesota)**

Institutional Affiliation: University of Veterinary & Animal Sciences, Lahore, Pakistan

1. **Shoaib Ashraf, DVM, PhD (McGill University)**

Institutional Affiliation: Massachusetts Hospital Harvard Medical School, Boston, USA

1. **Moneeb Ashraf, MBBS, M.Phil. (Federal Post-Graduate Medical Institute)**

Institutional Affiliation: Mayo Hospital, King Edward Medical University, Lahore, Pakistan

1. **Sohaib Ashraf, MBBS (Shaikh Khalifa Bin Zayed Al-Nahyan Medical & Dental College)**

Institutional Affiliation: Massachusetts Hospital Harvard Medical School, Boston, USA

**Co-Investigators:**

1. **Ali Ahmad, PhD (Virology)**

Institutional Affiliation: Department of Immunology & Virology, University of Montreal, Sainte Justin Hospital, Canada.

1. **Qazi Abdul Saboor, MBBS, FCPS (Cardiology)**

Institutional Affiliation: Department of Cardiology, Shaikh Zayed Medical Complex, Lahore, Pakistan

1. **Uzma Mamoon, MBBS, FCPS(Medicine)**

Institutional Affiliation: Department of Medicine, Shaikh Zayed Medical Complex, Lahore, Pakistan

1. **Muhammad Ahmad Imran, MBBS**

Institutional Affiliation: Microbiology Department, Shaikh Zayed Medical Complex, Lahore, Pakistan.

1. **Larab Kalsoom, MBBS**

Institutional Affiliation: Department of Medicine, Services Institute of Medical Sciences, Lahore, Pakistan

1. **Sundas Rafique, MBBS**

Institutional Affiliation: Department of Medicine, Mayo Hospital, King Edward Medical University, Lahore, Pakistan

1. **Iqra Farooq, MBBS**

Institutional Affiliation: Department of Pediatric Surgery, Children Hospital, Lahore, Pakistan.

**NATIONAL COLLABORATORS:**

1. **Dr. Umer Naveed, PhD**

Designation: Research Scholar in Parasitology & Pharmacology

Institutional Affiliation: Royal (Dick) School of Veterinary Studies and Roslin Institute, Scotland, UK

1. **Dr. Fazli Mabood, PhD**

Designation: Research Scientist Microbiology

Institutional Affiliation: CHU Sainte-Justine Hospital, University of Montreal, Canada

1. **Dr. Muhammad Bilal, M.Phil.**

Designation: Research Associate Virologist

Institutional Affiliation: McGill University, Sainte-Anne-de-Bellevue, Quebec, Canada;

**LEAD BIOSTATITICIAN:**

**Prof. Dr. Muhammad Azam, PhD**

Designation: Dean Faculty of Life Sciences Business Management, Professor of biostatistics, Lead Biostatistician

Institutional Affiliation: University of Veterinary & Animal Sciences, Pakistan

**RESEARCH PHARMACISTS & DRUG SAFETY PANELISTS:**

1. **Dr. Asad-ullah, Pharm.D, M.Phil.**

Designation: Assistant director Drug Regulation Authority Pakistan

Institutional Affiliation: Drug Regulation Aurthority Pakistan (DRAP)

1. **Dr Zaka-ur-Rehman, PhD**

Designation: Chief drug controller punjab

Institutional Affiliation: Ministry of Health Punjab, Pakistan

1. **Dr. Syed Muhammad Muneeb**, **PhD**

Designation: Assistant Professor of Pharmaceutical Sciences

Institutional Affiliation: Institute of Pharmaceutical Sciences, UVAS

1. **Dr. Faisal Nadeem, PhD**

Designation: Assistant Professor of Pharmaceutical Sciences

Institutional Affiliation: Institute of Pharmaceutical Sciences, UVAS

**CLINICAL EXPERT PANEL:**

- **Dr. Talha Mahmood, MD (Pulmonology)**

Designation: Professor Pulmonology

Institutional Affiliation: Shaikh Zayed Medical Complex, Lahore, Pakistan.

- **Dr. Qazi Abdul Saboor, FCPS(Cardiology)**

Designation: Professor Cardiology

Institutional Affiliation: Shaikh Zayed Medical Complex, Lahore, Pakistan.

- **Prof. Dr. Amber Malik, MRCP.**

Designation: Professor Cardiology

Institutional Affiliation: Evercare Hospital, Lahore, Pakistan

- **Prof. Dr. Imran Anwar, FCPS.**

Designation: Professor Surgery

Institutional Affiliation: Shaikh Zayed Medical Complex, Lahore, Pakistan

- **Dr Nighat Masood, FCPS (Medicine)**

Designation: Associate Professor of Medicine unit 2

Institutional Affiliation: Services Institute of Medical Sciences, Lahore, Pakistan.

**Abstract**

Background: Since the first report from Wuhan China in December 2019, coronavirus disease 2019 (COVID-19) caused by the novel coronavirus, while infecting millions globally. An effective response requires the development of a successful treatment regimen on an urgent basis while maintaining the clinical safety of patients. Despite a wide range of advised options for the treatment of COVID-19, a single strategy to tackle this pandemic remains elusive, thus far. That’s why we are conducting a clinical trial to find out the efficacy of iodine complex in treating the COVID-19 patients.

Objective: The objective of this study is to measure the effect of ionic-iodine polymer complex in treating the coronavirus disease 2019 (COVID-19) patients to clear a viral load of severe respiratory syndrome coronavirus-2 (SARS-CoV-2) along with a reduction in time taken to alleviate symptoms.

Method: The proposed study is a placebo-controlled, add-on, randomized trial using parallel group designs. This is a close-label and adaptive, multi-centered design with a 1:1:1:1 allocation ratio and superiority framework. It will be conducted in Shaikh Zayed Post-Graduate Medical Complex, Ali Clinic and Doctors Lounge, Lahore, Pakistan. This study will have three arms (50 patients in each) which will receive ionic-iodine polymer complex with 200mg of elemental iodine: interventional arm A will have encapsulated, arm B will receive suspension syrup form, arm C will get throat spray. while arm X will be standard care with placebo. Data will be collected on self-constructed, close-ended questionnaires after obtaining written consent. Data will be analyzed using SAS version 9.4. COVID-19 patients will be monitored by RT-PCR and HRCT (High-Resolution Computed Tomography) chest. In addition to these. the duration of the symptomatic phase and mortality benefits will be analyzed in both groups.

Discussion: The study is designed to measure the superior efficacy of the Iodine complex as an add-on in treating COVID-19 positive patients with mild to moderate symptoms. This combination is hypothesized to improve different parameters like rapid viral load reduction and a negative RT-PCR, rapid clinical progress, clear HRCT chests, low mortality rates, and reduction in hospitalization. The trial will aid in devising a better strategy to cope with COVID-19 in a relatively inexpensive and accessible way. The implications are global, and this could prove itself to be the most manageable intervention against COVID-19 especially for patients from limited-resource countries with deprived socioeconomic statuses.

Registration Number: Clinical Trial registration is NCT04473261

**Keywords**

Iodine, COVID-19, SARS-CoV-2

**Introduction**

**Background and rationale {6a}**

As the world was still recovering from The Great War, mayhem which shook the world and killed more than 40 million soldiers and civilians, mother earth suffered from a pandemic of Influenza. This deadly virus, in a year, spread everywhere on earth and diseased roughly 500 million and killed 100 million people. Surprisingly after the 101^st^ anniversary of that horrendous plague, we are again witnessing a fast-spreading, highly infectious severe respiratory syndrome coronavirus-2 (SARS-CoV-2 sweeping across the continents. This disease first originated in Wuhan, China in December 2019 which was later called coronavirus disease 2019 (COVID-19). The illness was declared a pandemic by the World Health Organization (WHO) in March 2020^(^[1](#_ENREF_1)^,^ [2](#_ENREF_2)^)^. Despite being huge data available on the virus epidemiology, pathophysiology, virology, diagnosis, prevention, and management still, most of the available management options are unable to provide promising results and this demands a dire need to find a cure for this highly contagious virus.

Despite this wide range of options available for the treatment of COVID-19 none has been proven to be a definitive therapy against this virus. This makes it the need of houthe r to think outside the box and prescribe newer formulations and conduct trails for treating COVID-19. This makes it the need of the hour that a novel idea of using micronutrients should also be proposed as an anti-viral in this trial.

Micronutrients are also essential for the body to produce enzymes, hormones, and other substances essential for proper growth and development .^(^[3](#_ENREF_3)^)^ Their deficiencies have been reported in many diseased states. Diet deficient in these nutrients may lead to compromised humoral and cell-mediated immunity. ^(^[4](#_ENREF_4)^)^ Iodine is considered an excellent antimicrobial and specifically antiviral action of the elements commonly used in nutrition. ([5-7](#_ENREF_5)) Cell culture studies also show that higher concentrations of iodine have exceptional antiviral activities. The potential of iodine against Human Immunodeficiency Virus (HIV) has been tested due to its powerful antiviral activity([8](#_ENREF_8)). It highlighted some important aspects of the mechanism of action of iodine as an antiviral agent([9](#_ENREF_9)). Moreover, through scientific studies, it has been established that iodine complexes circulate throughout the body in the extracellular fluids found between the cells of the body. If cell surface proteins have the amino acid tyrosine on the outside, the passing iodine complex reacts with this tyrosine. This reaction denatures the proteins and thus kills the abnormal cell. Intra membrane proteins may have tyrosine which is only exposed when the membrane is distorted by abnormal cell development. So, the iodine complex supports the surveillance system for removing abnormal cells from our bodies. Iodine complex inactivates viruses by interfering with the protein coat of the virus hence the ability of the viruses to adsorb to host cell is impaired. Iodine complex also triggers a mechanism for apoptosis (normal programmed death of cells as part of their life cycle) a process for destroying cells that present a threat to the integrity of the organism, like cells infected with viruses ([9](#_ENREF_9)).

Potassium iodide boosts not only the humoral immune system of the body by increasing immunoglobulin production but also increases peripheral lymphocytes level which is important in the host response against viruses. Supplementation of potassium iodide is essential for enhancing humoral immunity against pathogens([10](#_ENREF_10)).

In-vitro studies were conducted on the anti-viral properties of iodine complex against severe respiratory syndrome coronavirus-2 (SARS-CoV-2) at University of Veterinary and Animal Sciences, Lahore, Pakistan which showed strong to moderate antiviral activity ([11](#_ENREF_11)) Another in vitro trial has suggested the antiviral activity of Povidone-iodine against SARS-CoV-2 when exposed for more than 60 seconds and can be used in various concentrations in oral and nasal formulations ([12](#_ENREF_12)). Consistent with that is an in vitro study published in *JAMA* showed that the use of Povidone-iodine at different concentrations successfully deactivated the COVID-19 virus in only 15 seconds as compared to 70% ethanol used as control which failed to do so ([13](#_ENREF_13)). This was also observed in the past in SARS-CoV and Middle Eastern Respiratory Syndrome (MERS) in which iodine-containing preparations showed the same virucidal effect as 70% ethanol as control ([14](#_ENREF_14)).

With the rise of a second wave of coronavirus worldwide, it is considered to evaluate iodine complex as a potentially affordable and accessible remedy to mitigate infection.

**Objectives {7}**

The objective of the study is to measure the efficacy of the ionic-iodine polymer complex in reducing the length of the symptomatic phase along with earlier SARS CoV-2 clearance and radiologically better chest as compared to the placebo group.

**Trial design {8}**

This is a placebo-controlled, multi-armed, add-on, interventional, randomized trial using a parallel-group design. It is close-labeled, adaptive, and multi-centered with a 1:1:1:1 allocation ratio design with a superiority framework.

**Methods: Participants, interventions and outcomes**

**Study setting {9}**

Clinical sampling will be done from Shaikh Zayed Post-Graduate Medical Complex, Ali Clinic and Doctors Lounge, Lahore, Pakistan.

**Eligibility criteria {10}**

All COVID-19 diagnosed and declared patients presented in study settings during the study period, with mild to moderate disease severity.

Inclusion Criteria:

- Positive RT-PCR (Real-Time Polymerase Chain Reaction)
- Both genders and age 18 years and above

Exclusion Criteria:

- Presence of any co-morbidities like liver disease, thyroid dysfunction, ischemic heart disease, immuno-compromised patient, or any other chronic ailment.
- Females who are pregnant and breastfeeding
- If a patient is allergic to iodine.

**Who will take informed consent? {26a}**

The site investigator will take written informed consent from all trial participants by giving them the specifically constructed informed consent form.

**Additional consent provisions for collection and use of participant data and biological specimens {26b}**

Site investigators will be responsible for taking consent regarding every other matter if required. An informed consent form will contain a section on permission to draw and conduct specified tests on blood samples and conduct radiological investigations as per the protocol of the study.

**Interventions**

**Explanation for the choice of comparators {6b}**

All 4 arms will be receiving standard care as per the protocol of the study setting. Placebo comparator group, arm X, will receive a placebo empty capsule, in addition to standard care.

Interventional arms will be given ionic-iodine polymer complex (200mg of elemental iodine) in 3 different formulations as follows along with standard care.

- Arm A will be receiving iodine complex (capsule form)
- Arm B will be receiving iodine complex (syrup form)
- Arm C will be receiving iodine complex (nasal spray)
- Arm X will be placebo-control group

**Intervention description {11a}**
In this multi-armed study, ionic-iodine polymer complex will be given using three formulations with 200mg of elemental iodine to evaluate efficacy for a maximum of 14 days or when the patient is fully recovered.

- Arm A will be receiving 200mg iodine complex capsule three times a day
- Arm B will be receiving iodine complex suspension syrup form 40ml three times a day
- Arm C will be receiving iodine complex nasal spray 2 puffs three times a day.
- Arm X will be receiving a placebo of empty capsule three times a day.

All 4 arms will be receiving standard care as per version 3.0 of clinical management guidelines for COVID-19 established by the Ministry of National Health Services of Pakistan COVID-19 guidelines of the study setting.

**Criteria for discontinuing or modifying allocated interventions {11b**

Fixed doses will be given throughout the study and interventional drug administration will be stopped immediately in following conditions.

1. patient becomes severely symptomatic during trial conduction.
2. Any adverse drug reaction.
3. Organ failure secondary to any administered drug.
4. denies/backs off from further participation.

Regardless of any of these conditions, the participant’s data will be retained and analyzed in the trial to follow-up and prevent any loss of data.

**Strategies to improve adherence to interventions {11c}**

To improve adherence to the intervention, participants will be counselled about the advantages of this study. All the participants will be monitored regarding compliance to their assigned treatment strategy and health professionals will administer the drugs. As this trial will include quarantined patients, hence direct observational method will be sufficient to make sure compliance and adherence to the interventions.

**Relevant concomitant care permitted or prohibited during the trial {11d}**

As per hospital protocol (study setting), the care and interventions permitted will be used and no specific prohibited care is in this trial. As far as drug reactions are concerned, they will be treated by health care workers on the spot and will be reported afterwards.

**Provisions for post-trial care {30}**

No post-trial care will be needed in our study setup as half-lives of the administered drugs are within hours to days.

**Outcomes {12}**

Primary Outcomes

1. SARS-CoV-2 RT-PCR
2. HRCT chest score
3. Alleviation of symptoms

**RT-PCR** will be done on admission day (0 day) and then after every 4^th^ day for 12 days or till the symptoms are resolved and RT-PCR gets negative. RT-PCR will only be shown as positive or negative (as a limitation of our study of not getting the viral load).

**HRCT chests**

Like previous study ([15](#_ENREF_15)), a maximum of 4 HRCT chest will be performed starting from day zero followed by every fourth day till patients PCR become negative.

HRCT SCORING will be done as follows: Lungs divided into five lobes and each lobe is given 1 number. Total score is 25. Each lobe is scored from 0 to 5 as:

• 0 = no involvement

• 1 = <5% involvement

• 2 = 25% involvement

• 3 = 26%-49% involvement

• 4 = 50%-75% involvement

• 5 = >75% involvement

The individual lobar scores i.e., from 0 (no involvement) to 25 (maximum involvement) make up the total HRCT chest score ([16](#_ENREF_16)).

The HRCT findings are described via standard international terms, which are classified by the Fleischner Society glossary with peer-reviewed literature on viral pneumonia. The terms being used are ground glass opacity (GGO), crazy-paving pattern, and consolidation ([17](#_ENREF_17), [18](#_ENREF_18)).

**Time taken for the alleviation of symptoms:**

The number of days the patient remained symptomatic i.e., the difference between onset of COVID-19 symptoms to complete resolution.

**Secondary Outcomes:**

**Mortality:**

This criterion will tell us the number of patients who died after 30 days of treatment with iodine complex.

**Participant timeline {13}**

Schedule of enrolment, interventions (including any run-ins and washouts), assessments, and visits for participants have been shown in a schematic diagram (Figure 1).

**Sample size {14}**

50 patients in each arm with a total of 200 patients sample size for a multi-centered study in Pakistan.

**Recruitment {15}**

Recruitment will be done in Shaikh Zayed Medical Complex, Ali Clinic, and Doctors Lounge with mild to moderate symptomatic patients. All patients’ records will be analyzed at the center and eligible participants will be separated. All the eligible participants will be assessed according to our inclusion and exclusion criteria. Fully equipped site investigators with full precautions will do all of these proceedings. At the start of the study trial, all recruited participants have to provide a written informed consent form as per the plan to get them enrolled for the study.

**Assignment of interventions: allocation**

**Sequence generation {16a}**

Stratification for initial COVID-19 status (or days from initial symptoms as a proxy), age groups, gender and co-morbidities will be used to ensure that groups remain balanced in size for either arm after written informed consent to participate in our study. Randomization will be done using lottery method. As patients might be admitted at different times so they will be recruited after taking written informed consent (following all standard protocol for infection control and disinfection) and will be randomized by selecting a slip from the box containing 50 slips of each arm labeled as A, B, C and X. Arm A, B and C will be the add-on interventional arms while arm X will be the placebo arm.

**Concealment mechanism {16b}**

The allocation sequence will be computer generated that will be concealed from all site investigators, allocated participants and treatment providers until the final interventional allocation is done.

**Implementation {16c}**

The site investigators who will do the recruitment of interventional groups will request the principal investigator, Dr. Shoaib Ashraf (ShA) for randomization. The principal investigator (ShA) will send his answer form to the treatment providers in concealed envelops at allocated corona centres. The therapists will have no influential role in study outcomes and analysis while only disclosing the treatment plans to patients. Site investigators and other study members that are involved in participant’s enrolment will not be allowed to receive allocation information in order to prevent study bias.

**Assignment of interventions: Blinding**

**Who will be blinded {17a}**

Trial members including site investigators, outcome assessors and data analysts will be blinded. The details will be announced after locking the data in a database sheet at the end of the trial. Trial participants, care providers, outcome assessors and data analysts are blinded, respectively, by using placebo group, by using site investigators to provide placebo or drugs to participants, by using blinded clinicians to assess the clinical outcome and laboratory or radiological findings while by using analysts from other institution that are not having any conflict of interest in research while study chair being the only person knowing the participants allocated and analyzed in interventional arm.

In this study experimental drug and placebo will look alike, as they will have same packing but unique randomization codes. Participants, site investigators, care providers, outcome assessors, study coordinators, data managers, and statisticians will be blinded, and blinding codes will be revealed at the end of this study.

**Procedure for unblinding if needed {17b}**

Unblinding is permissible if the patient develops severe symptoms and needed extra treatment by revealing a participant’s allocated intervention during the trial. If unblinding is required, the trial managers and data coordinators will have access to group allocations and any unblinding will be reported.

**Data collection and management**

**Plans for assessment and collection of outcomes {18a}**

Microsoft Access, a database management system (DBMS) of Microsoft Office will be used to ensure the data safety. Two site investigators will enter the data, recheck twice for possible errors separately, and make certain its integrity. Principal investigators will visit twice weekly the study site while ethical committee will overview the study on weekly basis. Trial steering committee members will make unexpected and unplanned visits as well. There is no conflict of financial and non-financial interest with sponsors and researchers.

**Plans to promote participant retention and complete follow-up {18b}**

All the participants will be ensured their safety and will be guided about the study conduction and its beneficial outcomes. This study will be conducted till the patients hospitalized or home-quarantined in homes test negative for SARS-CoV-2 RNA in two consecutive nasopharyngeal swabs done using RT-PCR. All the data will be collected while the patient is already admitted in the hospital except 30-day mortality. Contact numbers and addresses of all participants will be reported at the start of study for which can be used, if needed, after written consent from the patient. Follow up will be done using phone numbers of the patients to access the mortality benefit of intervention.

**Data management {19}**

Participants IDs will be used for confidentiality purposes and these IDs will be linked to demographic information securely and separately. The final data set of RCT will have coded data and can only be assessed by principal investigators. All outcomes will be double-checked by the researchers prior to data collection and data storage. To ensure data’s integrity and safety various meetings by the research team will be conducted on regular basis.

**Confidentiality {27}**

In addition, confidentiality of participants’ data is ensured by using participants’ IDs rather than identifiable information in the dataset (i.e. coding) and by storing the document linking the IDs to the identifiable information separately and securely.

**Plans for collection, laboratory evaluation and storage of biological specimens for genetic or molecular analysis in this trial/future use {33}**

Trained staff will collect nasopharyngeal swabs samples as per biosafety and personal safety guidelines of World Health Organization. Samples will be maintained at -80 degrees. Patient’s follow up will be done daily by the investigators while PCR will be repeated every 4^th^ day for monitoring of primary end point (measuring time to return for COVID19 RT-PCR test to return negative). Patients will be evaluated clinically on daily basis; relevant investigations will be repeated as needed.

**Parameters to be checked Daily:** CBC, LFTs, RFTs, Serum Electrolytes, spO2, ABGs, ECG, cardiac profile.

**Parameters to be on alternate Days:** LDH, Serum Iron, Serum Ferratin, TIBC.

**Parameters to be checked every 4^th^ Day:** ESR, CRP, TSH, IL-6, IL-10, coagulation profile, D-Dimers, heme, Procalcitonin, RT-PCR Test, HRCT Chest, Echo (using Butterfly IQ).

**Parameters to be checked at the 19^th^ day of study:** IgG antibody level against SARS-CoV-2

**Statistical methods**

**Statistical methods for primary and secondary outcomes {20a}**

Mean ± S.D will be used for quantitative data and f(%) will be used for categorical data. Frequency and percentages will be measured for categorical data. Data normality will be checked using Shapiro Wilks test., if data is normal independent sample t-test will be used to compare quantitative outcome such as mean hospital stay otherwise Mann Whitney U test will be used to compare median of these quantitative data. Chi-square t-test/ Fisher’s Exact test will be applied to compare severity of symptoms and outcome (discharge or mortality), etc. For follow up analysis Wilcoxon test will be applied. If data supports the necessary assumptions of time to event data, survival analysis/ Kaplan Meier test will be applied. P-value ≤ 0.05 will be considered as significant.

| **Outcome** | **Hypothesis** | **Outcome measure** | **Method of analysis** |
| --- | --- | --- | --- |
| **PRIMARY** | | | |
| 1. RT-PCR  (every 4^th^ day) | earlier RT-PCR negative | Positive/ negative | Comparison of proportion every 4^th^ day using Z-Test  Test for measurement of association using Chi-Square Test |
| 2. HRCT Chest  (every 4^th^ day) | improvement in HRCT chest | Lungs divided into five lobes and each lobe is given 1 number. Total score is 25. Each lobe is scored from 0 to 5 as:  0 = no involvement  1 = <5% involvement  2 = 25% involvement  3 = 26%-49% involvement  4 = 50%-75% involvement  5 = >75% involvement | Repeated measured design will be applied using ANOVA  T-Test will be applied to make comparison at 4^th^ day |
| 3. Time to alleviation of symptoms | Earlier resolution of symptoms | Symptomatic duration in days | Univariate analysis log rank test |
| **SECONDARY** | | | |
| 4. Mortality | Decrease in mortality | Yes/No | Fisher Exact Test |

**Interim analyses {21b}**

The risk aptitude for this study is customized as low risk, considering the use of nutraceutical product. However, as it involves vulnerable COVID-19 patients and novel drugs are being tested for repurposing strict safety measurements will be taken. As a part of our safety measurements the co-investigator, Dr Qazi Abdul Saboor (Professor of Cardiology, SZH, Lahore), with the biostatistician, Prof. Dr. Muhammad Azam (Dean faculty of biostatistics, UVAS, Lahore) will conduct an interim analysis. The prime focus of this analysis will be mortality and incidence of any serious adverse effects. This will be carried out after randomization and inclusion of half of the patients. For these specific outcomes, those conducting the interim analysis will be unblinded. No stopping rules for the primary endpoint have been defined as this is the first trial of its kind.

**Methods for additional analyses (e.g. subgroup analyses) {20b}**

Adjusted and subgroups analysis may be applied as per the biostatistician, if needed. In that case both unadjusted and adjusted analyses are provided along with the main analysis

**Methods in analysis to handle protocol non-adherence and any statistical methods to handle missing data {20c}**

The intention-to-treat analysis set will be used to test the superiority. All patients will be considered as randomized despite receiving the randomized treatment as per our anticipation. Reasons for each group’s randomization and withdrawal will be reported and compared qualitatively and sensitivity analysis (augmented data) is being used to overcome the effect of any missing data on results. The participants who withdraw consent for continued follow-up (Dropouts) will be assessed by modern imputation methods for missing data

A set of clinically reasonable imputations will be created for each dropout and it will tell us about the respective outcome. For this purpose, a set of repeated imputations will be used and this will be created by predictive models based on the majority of participants with complete data. The ambiguity in the modelling process will be reflected by the imputation models along with the inherent variability in patient outcomes, as evident by the complete data.

Analysis will be conducted for each imputed-and-completed dataset after the completion and compilation (collection) of all the data i.e. complete and imputed. To estimate the effectiveness of the treatment Rubin’s method of multiple (i.e., repeated) imputation will be used. We propose to use 15 datasets (an odd number to allow use of one of the datasets to represent the median analytic result).

**Plans to give access to the full protocol, participant level-data and statistical code {31c}**

Only the principal investigator, Prof. Dr. Muhammad Ashraf (MAs) will have access to the full trial dataset in order to ensure that the overall results are not disclosed by an individual of trial prior to the main publication. Grant public access to the full protocol, participant-level dataset, and statistical code will be given through clinicaltrials.gov.

**Oversight and monitoring**

**Composition of the coordinating centre and trial steering committee {5d}**

Coordinating center and trial steering committee will be comprised of medical lab technologist (Sidra Ashraf, PhD), clinical pharmacist (Faisal Nadeem, PhD), clinical pharmacologists and toxicologist (Moneeb Ashraf, PhD), virologist (Mateen Izhar, PhD), immunologist (Ali Ahmad), biostatisticians (Muhammad Azam, PhD), public health expert (Usman Iqbal, PhD), epidemiologist (Ayesha Humayun, PhD), ethical expert (Muhammad Suhail, M.Phil) and consultants of medicine (Dr. Uzma, MBBS), pulmonology (Talha Mahmud, MD) and cardiologist (Amber Malik, MBBS). This committee will be responsible for the safety, trial safety and dosage calculation to get results of endpoints. This will have all the authority to stop the clinical trial all together. Data management team will be comprised of principal investigators, co-investigators and site investigators. This team will be responsible to ensure the execution of the clinical trial in best possible way as defined by the study protocol. Site investigators are responsible for data collection and quality check of data at collection points/ study setting. Kiwan Akram will manage data compilation, cleaning, editing, and entry on SAS along with Biostatistician.

**Composition of the data monitoring committee, its role and reporting structure {21a}**

Data monitoring committee (DMC) has a biostatistician (Muhammad Azam, PhD), an epidemiologist (Ayesha Humayun, PhD), and public health expert (Usman Iqbal, PhD), a microbiologist (Mateen Izhar, PhD), a pulmonologist (Talha Mehmood, MD) and principal investigators (Muhammad Ashraf, PhD and Shoaib Ashraf, PhD) in it. There is no financial or non-financial conflict of interest as the committee will be independent of sponsor and competing interests.

**Adverse event reporting and harms {22}**

The researchers will record any adverse, unpredictable or undesirable sign and symptom and it will be discussed with the care providers. A comprehensive evaluation will be conducted to evaluate the co-relation between experimental drug and the developing signs and symptoms. The investigator will respond appropriately to ensure the wellbeing of the patient in case of any unforeseen event and all the details will be written carefully. Moreover, regular follow-up will be made certain until the patient regains his/her health. If the adverse event happens during the study intervention will be reported Institutional Review Board (IRB).

**Frequency and plans for auditing trial conduct {23}**

Weekly audit will be done by principal investigators. Monitors will audit by visiting trial sites while performing and resolving solutions to various problems.  The monitor will verify the following variables for all patients on every visit: biodata of participants, signed informed consent, eligibility criteria, date of randomization, group allocation, treatment assigned and adverse events if any. Auditing of the clinical trial will be done by trail steering committee in weekly zoom meeting where all the audit will be provided by the site investigators and research coordinators.

**Plans for communicating important protocol amendments to relevant parties (e.g. trial participants, ethical committees) {25}**

In order to modify protocols including eligibility criteria and outcomes, permission will be needed to get approved by the trial steering committee and the notification will be done to relevant parties including IRB trial registry. All the plans about any amendments in our trial will be communicated to the trial site staff in person.

**Dissemination plans {31a}**

The publications subcommittee will review the publication, all the endpoint data, primary outcome analysis and the study results and recommend the changes to the author. After the changes being done it will finally submit its recommendations to the steering committee for approval. Study results will be disclosed to all study participants, member physicians, patients and other medical personnel.

**Discussion**

Although the vaccine is around the corner, a year has passed yet no cure for COVID-19 is available and most of the treatment relies on supportive measure. Here the role of PVP-1 comes, as it solutions have been long used as microbicidal agents and they offer appropriate safety profile ([19](#_ENREF_19)). Our study shows that patients treated with oral formulations of iodine show better prognosis than the placebo hence establishing its role in treating the disease. It has also been hypothesized that less number of deaths seen in Japan despite boosting a large number of old age population is because of role of iodine in supporting innate immunity against viral pathogens since Japanese are famous for taking higher amounts of iodine ([20](#_ENREF_20)) Since different preparations have different concentration dependent efficacy and side effects this will provide us to a better idea of optimal dosage with better efficacy and limited toxicity. The efficacy of iodine complex which will be reported in our trial will be compared to those of other topical alternates used to stop viral growth in nasopharynx as published in literature to establish the superiority, if any, of the iodine-based preparations. As previous studies have some concentration dependent damage by iodine complexes to normal mucociliary mechanism and nasal epithelium ([21](#_ENREF_21)) ([22](#_ENREF_22)) hence this study will look for such possible adverse outcomes keeping regular follow. Possible lowering of efficacy of iodine preparation will be looked for as nasal secretions, debris and poor nasal hygiene dilute the concentration of the formulations and reduce its penetrance. The study will be limited to viral load clearance rather than measuring viral load reduction. **Trial status**

Original protocol dated: 01/07/2020; Version: 1.0

Patient recruitment started in July’20 and will be completed till July’21

**Abbreviations**

| **Abbreviation** | **Full Form** |
| --- | --- |
| COVID-19 | coronavirus disease 2019 |
| DM | Diabetes Mellitus |
| DNA | Deoxyribose Nucleic Acid |
| FAIR | Findable, Accessible, Interoperable, Re-usable |
| HRCT | High Resolution Computerized Tomography |
| HTN | Hypertension |
| IRB | Institutional Review Board |
| RT-PCR | Real time polymerase chain reaction |
| RNA | Ribose Nucleic Acid |
| SARS-CoV | Severe acute respiratory syndrome coronavirus |
| SARS-CoV-2 | Severe acute respiratory syndrome coronavirus 2 |
| SZH | Shaikh Zayed Hospital |
| UVAS | University of Veterinary & Animal Sciences |
| KEMU | King Edward Medical University |
| SZPGMI | Shaikh Zayed Post-Graduate Medical Institute |
| PVP-1 | Povidone-iodine 1 |

**Declarations**

**Acknowledgements:**

The authors would like to show gratitude to the following colleagues who provided moral support and intellectual inputs in designing this trial: Dr. Asad-ullah, Assistant Director Drug Regulation Authority Pakistan, Dr. Zaka-ur-Rehman, PhD, Chief Drug Controller Punjab.

**Funding {30}**

Funding will be provided by Shaikh Zayed Post-Graduate Medical Complex, Lahore, Pakistan.

**Availability of data and materials {29}**

The datasets used or analysed during the current study will be available from the corresponding author upon reasonable request.

**Ethics approval and consent to participate {24}**

Ethical approval has been applied for authorization to institutional review board of Shaikh Zayed Hospital, Lahore, PK and later on to Services Institute Medical Sciences, Lahore, PK. Written, informed consent to participate will be obtained from all participants.

**Competing interests {28}**

The authors affirm that they have no competing interests and according to the standards of scientific integrity the publication of both positive and negative study results will be ensured. All the study data (if reasonable) will be made accessible guided by the FAIR principles along with the perspective of relevant laws and privacy regulations. Authorship eligibility follows conventional academic standards. No professional writers had been involved in this.

**CONSORT 2010 Flow Diagram**

PCR positive for COVID-19

## Enrollment

Randomized

All patients eligible for study using enrollment criteria

Excluded (n= )

♦ Not meeting inclusion criteria (n= )

♦ Declined to participate (n= )

♦ Other reasons (n= )

**Allocated to experimental group** (n=)

♦ Received allocated intervention (n= )

♦ Did not receive (give reasons) (n= )

**Allocated to standard therapy** (n=)

♦ Received standard care (n= )

♦ Did not receive allocated intervention (give reasons) (n= )

## Allocation

Lost to follow-up (give reasons) (n= )

Discontinued intervention (give reasons) (n= )

Lost to follow-up (give reasons) (n= )

Discontinued intervention (give reasons) (n= )

## Follow-Up

Analyzed for response, on day PCR gets negative (n= )
♦ Excluded from analysis (give reasons) (n= )

Analyzed for response, on day PCR gets negative (n= )
♦ Excluded from analysis (give reasons) (n= )

## Analysis

Determination of days PCR has taken to be negative in previously positive patients where standard therapy was given alone along with a better HRCT score and alleviation of disease symptoms

Determination of days PCR has taken to be negative in previously positive patients where intervention was given along with a better HRCT score and alleviation of disease symptoms

**FIGURE 1**

|  | **STUDY PERIOD** | | | | | | | | |
| --- | --- | --- | --- | --- | --- | --- | --- | --- | --- |
|  | **Enrolment** | **Allocation** | **Post-allocation** | | | | | **Discharge** | |
| **TIMEPOINT** | ***Day 0*** | ***Day 0*** | ***Day 0*** | ***Day 4*** | ***Day 8*** | ***Day 12*** | ***Day 16*** | ***Day X*** |  |
| **ENROLMENT:** |  |  |  |  |  |  |  |  |  |
| **Eligibility screen** | X |  |  |  |  |  |  |  |  |
| **Informed consent** | X |  |  |  |  |  |  |  |  |
| **Demographic details** | X |  |  |  |  |  |  |  |  |
| **INTERVENTIONS:** | | | | | | | | | |
| *[*Iodine Complex (Capsule)*]* |  |  |  |  |  |  |  |  |  |
| *[*Iodine Complex (Syrup)*]* |  |  |  |  |  |  |  |  |  |
| *[*Iodine Complex (Spray)*]* |  |  |  |  |  |  |  |  |  |
| *[Placebo}* |  |  |  |  |  |  |  |  |  |
| **ASSESSMENTS** | | | | | | | | | |
| *[*Routine Investigations*]* | X |  |  |  |  |  |  |  |  |
| *[RT-PCR]* | X |  | X | X | X | X | X |  |  |
| *[HRCT Score]* | X |  | X | X | X | X | X |  |  |
| *[Severity of Symptoms]* |  |  |  |  |  |  |  |  |  |
| *[Mortality]* |  |  |  |  |  |  |  | X |  |
| *[Adverse Effects]* |  |  |  |  |  |  |  |  |  |

**FIGURE 2 : Distribution of patients in multiple arms**

**Informed Consent Form {32}**

This consent form addresses the participants of the clinical trial.

The title of our research project is: Efficacy Iodine Complex in Mild to Moderate COVID-19 patients (I-COVID-PK)

**Name of Principal Investigator**: Dr. Sohaib Ashraf

**Name of Organization**: Federal Post Graduate Medical Institute, Shaikh Zayed Medical Complex Lahore

This Informed Consent Form has two parts:

• Information Sheet (to share information about the research with you)

• Certificate of Consent (for signatures if you agree to take part)

**PART I:**

**Introduction:**

I am ________________________. Our team is conducting a research on COVID-19, which is a global health concern. I am providing you with all the necessary information and invite you to be part of this research. Before making any decision, whether you will participate or not, you may take your time and talk to someone you feel comfortable with about the research. If you have any difficulty in understanding anything, you may stop me as I go through the information and I will take time to explain. If you have questions later, you can ask them of me, the study doctor or the staff. A copy of the full informed consent form will also be provided to you.

**EXPLANATION:**

The proposed study is a placebo-controlled, add-on, randomized trial using parallel group designs. This is a close-label and adaptive, multi-centered design with 1:1:1:1 allocation ratio and superiority framework. It will be conducted in multiple designated corona centers established by the Government of Pakistan. This study will have four arms (50 patients in each): 3 experimental arms while one placebo group. All will be provided with standard care therapy. Data will be collected on self-constructed, close-ended questionnaires after obtaining written consent. Data will be analyzed using SAS version 9.4. COVID-19 patients will be monitored by RT-PCR and HRCT (High Resolution Computed Tomography) chest. In addition to these clinical improvements, duration of hospital stay, and mortality benefits will be analyzed in both groups. The trial will aid in devising a better strategy to cope with COVID-19 in a relatively inexpensive and accessible. The implications are global, and this could prove itself to be the most manageable intervention against COVID-19 especially for patients from limited-resource countries with deprived socioeconomic statuses.

**VOLUNTARY PARTICIPATION:**

It’s a voluntary participation in this trial. Whether you choose to be a part of it or not, all the services you receive at this hospital will continue and nothing will change. If you choose to participate you will have the authority to change your mind later and stop participating even if you agreed earlier.

**PROCEDURES AND PROTOCOL**

Participants in one group will be given the experimental treatment along with the standard treatment while participants in the other group will only be given the standard treatment as per hospital protocol. The healthcare workers will be monitoring you and the other volunteers’ vigilantly during the study. If there is anything you are anxious about or that is troubling you about the research, please talk to me or one of the other colleagues. For the purpose of this study, we will be with drawing 10ml of your blood (when needed), which will help us access your clinical laboratory data. Blood will be withdrawn from your arm, via trained staff, using a syringe through arterial/venous site.

You will be given nothing to take part in this research and confidentiality will be maintained. Identity of those taking part in this trial will not be revealed. The personal information that we collect from this research project will be kept confidential. Your personal information will be given numbers instead of your names. Only the researchers will be aware of the assigned number and we will lock that information up with a lock and key. This information can only be accessed by study director and study chair.

**SHARING THE RESULTS:**

Prior to making the knowledge, we get from this research, publicly available to the outside world, it will be shared with the participants through community/zoom meetings. Confidential information will not be shared. After these meetings, we will publish the results so that the knowledge gained can be shared with rest of the world.

**RIGHT TO REFUSE OR WITHDRAW:**

You do not have to take part in this research if you do not wish to do so. You may also stop participating in the research at any time you choose. It is your choice, and all of your rights will still be respected. Alternatives to participating if you do not wish to take part in the research, you will be provided with the established standard treatment available at the center/institute/hospital.

**Whom to Contact:**

If you have any questions you may ask them now or later, even after the study has started. If you wish to ask questions later, you may contact any of the following:

Dr. Sohaib Ashraf +923334474523

Dr Ahmad Imran +923338110708

Dr Moneeb Ashraf +923334461038

This proposal has been reviewed and approved by ethical review board of Shaikh Zayed medical complex, Lahore, which is a committee whose task it is to make sure that research participants are protected from harm. You can ask me any more questions about any part of the research study, if you wish to. Do you have any questions?

**PART II: Certificate of Consent**

I have read the foregoing information, or it has been read to me. I have had the opportunity to ask questions about it and any questions that I have asked, I have been answered to my satisfaction. I consent voluntarily to participate as a participant in this research.

Print Name of Participant__________________

Signature of Participant ___________________

Date ___________________________ Day/month/year

**If Illiterate (consent form being read to the witness)**

I have witnessed the accurate reading of the consent form to the potential participant, and the individual has had the opportunity to ask questions. I confirm that the individual has given consent freely.

Print name of witness_____________________

Thumb print of participant/ Signature of witness ______________________

Date ________________________ Day/month/year

I have accurately read out the information sheet to the potential participant, and to the best of my ability made sure that the participant understands.

I confirm that the participant was given an opportunity to ask questions about the study, and all the questions asked by the participant have been answered correctly and to the best of my ability. I confirm that the individual has not been coerced into giving consent, and the consent has been given freely and voluntarily.

A copy of this ICF has been provided to the participant.

Name of the person taking the consent ________________________

Signature of Researcher /person taking the consent__________________________

Date ___________________________ Day/month/year

**PERFORMA**

Medical Record #: ____________ ID: ___________

Name (optional): _____________ Age: _______ Gender: M / F

Domicile: ___________________ Oxygen Saturation: ___________

Study Center: ____________ Reference Doctor: _________

Contact Number: ________ Profession: _____________

Contact History: ______________ Blood Group: _____

Fever: __________ Fatigue: _______ Dry Cough: __________

SOB: ___________ Anorexia: ______ Sputum production: ____

Pharyngitis: ______ Myalgia: _______ GI disturbances: _______

Onset of symptoms: ____________ Resolution of symptoms: ____________

CBC: ____________________________________________________________________

RFTs: ____________________________________________________________________

LFTs: ____________________________________________________________________

ECG: _____________________________________________________________________

Chest X-ray: _______________________________________________________________

Clinical Course of Treatment:

____________________________________________________________________________________________

Clinical Data:

____________________________________________________________________________________________

| Day | ESR | CRP | IL-6 | IL-10 | PT, aPTT, INR | Heme | Pro-Calcitonin | RT-PCR | HRCT | Echo |
| --- | --- | --- | --- | --- | --- | --- | --- | --- | --- | --- |
| 0 |  |  |  |  |  |  |  |  |  |  |
| 4 |  |  |  |  |  |  |  |  |  |  |
| 8 |  |  |  |  |  |  |  |  |  |  |
| 12 |  |  |  |  |  |  |  |  |  |  |

| **Symptom** | Not Sick | Very Mild | Mild | | Moderate | | Severe | |
| --- | --- | --- | --- | --- | --- | --- | --- | --- |
|  | 0 | 1 | 2 | 3 | 4 | 5 | 6 | 7 |
| How Sick do you feel today |  |  |  |  |  |  |  |  |
| Fever |  |  |  |  |  |  |  |  |
| Cough |  |  |  |  |  |  |  |  |
| Sputum |  |  |  |  |  |  |  |  |
| Headache |  |  |  |  |  |  |  |  |
| Sneezing |  |  |  |  |  |  |  |  |
| Aches & Pains |  |  |  |  |  |  |  |  |
| Nausea |  |  |  |  |  |  |  |  |
| Fatigue |  |  |  |  |  |  |  |  |
| Running Nose |  |  |  |  |  |  |  |  |
| Sore Throat |  |  |  |  |  |  |  |  |
| Chills & Rigors |  |  |  |  |  |  |  |  |
| Shortness of Breath |  |  |  |  |  |  |  |  |
| GI disturbance |  |  |  |  |  |  |  |  |
| Loss of smell |  |  |  |  |  |  |  |  |
| Change of taste |  |  |  |  |  |  |  |  |
| Insomnia |  |  |  |  |  |  |  |  |
| Malaise |  |  |  |  |  |  |  |  |

Day of enrollment: __________ Date: _________ ID: _________

Please rate the average severity of your corona symptoms over the last 24 hours for each symptom:

**REFERENCES :**

1. (WHO) WhO. WHO situation report on coronavirus disease (COVID-19). 12 March 2020.

2. Organization WH. Coronavirus disease 2019 (COVID-19): situation report, 72. 2020.

3. Arigony AL, de Oliveira IM, Machado M, Bordin DL, Bergter L, Pra D, et al. The influence of micronutrients in cell culture: a reflection on viability and genomic stability. Biomed Res Int. 2013;2013:597282.

4. Balázs C, Leövey A, Szabó M, Bakó G. Stimulating effect of triiodothyronine on cell-mediated immunity. European journal of clinical pharmacology. 1980;17(1):19-23.

5. Assis D, Lim J. New pharmacotherapy for hepatitis C. Clinical Pharmacology & Therapeutics. 2012;92(3):294-305.

6. Harika R, Faber M, Samuel F, Kimiywe J, Mulugeta A, Eilander A. Micronutrient Status and Dietary Intake of Iron, Vitamin A, Iodine, Folate and Zinc in Women of Reproductive Age and Pregnant Women in Ethiopia, Kenya, Nigeria and South Africa: A Systematic Review of Data from 2005 to 2015. Nutrients. 2017;9(10).

7. Shivakoti R, Christian P, Yang W-T, Gupte N, Mwelase N, Kanyama C, et al. Prevalence and risk factors of micronutrient deficiencies pre-and post-antiretroviral therapy (ART) among a diverse multicountry cohort of HIV-infected adults. Clinical nutrition. 2016;35(1):183-9.

8. Mamo JC, Naissides M. Could iodine be effective in the treatment of human immunodeficiency virus and AIDS-associated opportunistic infections? Int J Infect Dis. 2005;9(5):292-3.

9. Sriwilaijaroen N, Wilairat P, Hiramatsu H, Takahashi T, Suzuki T, Ito M, et al. Mechanisms of the action of povidone-iodine against human and avian influenza A viruses: its effects on hemagglutination and sialidase activities. Virology journal. 2009;6(1):124.

10. Wilson R, Mc Killop J, Thomson J. The effect of pre-operative potassium iodide therapy on antibody production. European Journal of Endocrinology. 1990;123(5):531-4.

11. Altaf I, Nadeem MF, Hussain N, Nawaz M, Raza S, Ali MA, et al. An <em>in vitro</em> assessment of anti-SARS-CoV-2 activity of oral preparations of iodine complexes (RENESSANS). 2020:2020.06.29.171173.

12. Pelletier J, Tessema B, Westover J, Frank S, Brown S, Capriotti J. In Vitro Efficacy of Povidone-Iodine Nasal And Oral Antiseptic Preparations Against Severe Acute Respiratory Syndrome-Coronavirus 2 (SARS-CoV-2). 2020:2020.05.25.20110239.

13. Frank S, Brown SM, Capriotti JA, Westover JB, Pelletier JS, Tessema B. In Vitro Efficacy of a Povidone-Iodine Nasal Antiseptic for Rapid Inactivation of SARS-CoV-2. JAMA Otolaryngology–Head & Neck Surgery. 2020;146(11):1054-8.

14. Kariwa H, Fujii N, Takashima I. Inactivation of SARS coronavirus by means of povidone-iodine, physical conditions and chemical reagents. Dermatology. 2006;212 Suppl 1(Suppl 1):119-23.

15. Pan F, Ye T, Sun P, Gui S, Liang B, Li L, et al. Time course of lung changes on chest CT during recovery from 2019 novel coronavirus (COVID-19) pneumonia. Radiology. 2020:200370.

16. Francone M, Iafrate F, Masci GM, Coco S, Cilia F, Manganaro L, et al. Chest CT score in COVID-19 patients: correlation with disease severity and short-term prognosis. Eur Radiol. 2020;30(12):6808-17.

17. Franquet T. Imaging of pulmonary viral pneumonia. Radiology. 2011;260(1):18-39.

18. Koo HJ, Lim S, Choe J, Choi S-H, Sung H, Do K-H. Radiographic and CT features of viral pneumonia. Radiographics. 2018;38(3):719-39.

19. Eggers M, Koburger-Janssen T, Eickmann M, Zorn JJId, therapy. In vitro bactericidal and virucidal efficacy of povidone-iodine gargle/mouthwash against respiratory and oral tract pathogens. 2018;7(2):249-59.

20. Verheesen RH, Traksel RAM. Iodine, a preventive and curative agent in the COVID-19 pandemic? Med Hypotheses. 2020;144:109860-.

21. Reimer K, Wichelhaus TA, Schäfer V, Rudolph P, Kramer A, Wutzler P, et al. Antimicrobial effectiveness of povidone-iodine and consequences for new application areas. Dermatology. 2002;204 Suppl 1:114-20.

22. Kim JH, Rimmer J, Mrad N, Ahmadzada S, Harvey RJ. Betadine has a ciliotoxic effect on ciliated human respiratory cells. The Journal of laryngology and otology. 2015;129 Suppl 1:S45-50.
